# Effects of air pollution and meteorological factors on hematological exacerbation phenotypes in patients presenting to the emergency department with COPD exacerbation

**DOI:** 10.1007/s00484-026-03247-9

**Published:** 2026-06-09

**Authors:** Erdal Karataş, Nurettin Özgür Doğan, Sibel Balcı, İbrahim Ulaş Özturan, Elif Yaka, Serkan Yılmaz, Murat Pekdemir

**Affiliations:** 1 Dept. of Emergency Medicine, Kocaeli Derince Training and Research Hospital, Attending Physician, Kocaeli, Turkey; 2 Faculty of Medicine, Dept. of Emergency Medicine, Kocaeli University, 41001 Kocaeli, Turkey; 3 Faculty of Medicine, Dept. of Biostatistics, Marmara University, Istanbul, Turkey

**Keywords:** COPD exacerbation, Eosinophilic exacerbation, Hematological phenotype, Air pollution, Meteorological factors

## Abstract

To evaluate the short-term effects and lagged temporal patterns of air pollutants and meteorological factors on hematological exacerbation phenotypes in patients presenting to the emergency department with chronic obstructive pulmonary disease (COPD) exacerbation. Patients who presented to a tertiary emergency department with COPD exacerbation between April 2022 and April 2023 were retrospectively reviewed. Exacerbations were classified as eosinophilic, neutrophilic, or mixed-type based on admission complete blood counts. Daily air pollutant concentrations (PM₁₀, PM₂.₅, SO₂, NO₂, CO, O₃) and meteorological parameters were obtained from official monitoring stations. Day-level associations between environmental variables and phenotype-specific daily exacerbation counts were analyzed using quasi-Poisson distributed lag non-linear models (DLNM) and a time-stratified case-crossover design (lag 0–3 days). Of all exacerbations (n = 354), 65.8% were neutrophilic, 20.3% mixed-type, and 13.8% eosinophilic. The neutrophilic phenotype was associated with the most severe clinical presentation. No significant environmental association was identified for the overall COPD exacerbation count. In the eosinophilic phenotype, same-day exposure to CO and SO₂ was associated with increased exacerbation risk (RR = 1.39, 95% CI: 1.06–1.82 and RR = 1.21, 95% CI: 1.03–1.43, respectively). In the mixed-type phenotype, higher humidity was positively associated with exacerbation counts, whereas lower wind speed was associated with decreased counts. In the neutrophilic phenotype, an inverse association with O₃ observed in the DLNM was not confirmed in the case-crossover analysis. COPD exacerbations do not appear homogeneous with respect to environmental triggers; the eosinophilic phenotype showed clearer associations with gaseous pollutants, whereas the mixed-type phenotype showed associations with meteorological conditions. The absence of associations in the overall population supports the value of phenotype-based environmental risk assessment. These findings may contribute to the development of phenotype-oriented environmental warning strategies in COPD management.

**Supplementary Information:**

The online version contains supplementary material available at 10.1007/s00484-026-03247-9.

## Introduction

Chronic obstructive pulmonary disease (COPD) is characterized by persistent and generally progressive airflow limitation and is one of the leading causes of morbidity and mortality worldwide, imposing a substantial burden on health systems (GOLD 2026). COPD exacerbations are clinical events characterized by acute worsening of respiratory symptoms during the course of the disease and are associated with emergency department (ED) visits, hospitalizations, impaired quality of life, accelerated decline in pulmonary function, and increased mortality (Doğan et al. 2021).

Major triggers of COPD exacerbations include infections, tobacco exposure, air pollution, and meteorological changes (Hogea et al. 2020; Li et al. 2016; Javorac et al. 2021). In particular, short-term exposure to air pollution has been reported to increase exacerbation risk and rates of ED visits and hospitalizations; particulate matter (PM₁₀, PM₂.₅), nitrogen dioxide (NO₂), sulfur dioxide (SO₂), carbon monoxide (CO), and ozone (O₃) have been shown to worsen COPD symptoms (GOLD 2026; Li et al. 2016). Meteorological variables such as temperature, humidity, atmospheric pressure, and wind speed have also been shown to influence COPD exacerbations (Javorac et al. 2021).

In recent years, it has become increasingly recognized that COPD exacerbations are not a uniform clinical entity but rather a heterogeneous group of subtypes reflecting different inflammatory biology. In this context, classification of exacerbations according to hematological phenotypes (eosinophilic, neutrophilic, and mixed-type) has gained clinical importance and provides valuable information for prognostic assessment and personalized treatment strategies (Doğan et al. 2021; Kandemir et al. 2021). The eosinophilic phenotype has been associated with type-2 airway inflammation and may exhibit increased susceptibility to specific environmental exposures, whereas the neutrophilic phenotype has been more strongly linked to infections and innate immune responses (David et al. 2021; Narendra and Hanania 2019; Nurhussien et al. 2022; Saeed et al. 2024). The mixed phenotype, in which both inflammatory pathways are concurrently activated, represents a less well-characterized subgroup.

Although the effects of air pollution and meteorological factors on COPD exacerbations have been widely investigated, evidence on the differential effects of these environmental exposures across hematological exacerbation phenotypes remains limited. Studies examining phenotype-specific effects of short-term environmental variables in heavily industrialized regions are particularly scarce.

The aim of this study was to evaluate the effects and temporal patterns of air pollutants and meteorological factors on hematological exacerbation phenotypes (eosinophilic, neutrophilic, and mixed-type) in patients presenting with COPD exacerbation to a tertiary ED located in a region with intense industrial activity.

## Materials and methods

### Study design and setting

This is a single-center, retrospective observational study whose primary analytical component is an ecological time-series analysis examining the relationship between day-level environmental exposures and COPD exacerbation counts, complemented by patient-level hematological phenotyping. The design comprised two complementary components: (1) patient-level clinical and hematological phenotype characterization of COPD exacerbations (descriptive component), and (2) a day-level ecological time-series analysis of environmental exposures (analytical component).

The study was conducted in the adult ED of a university hospital between April 2022 and April 2023. The hospital is a tertiary referral center serving approximately 70,000 adult ED visits per year and is located in one of Turkey’s heavily industrialized regions. Air pollution data were obtained from the nearest official monitoring station of the Ministry of Environment, Urbanization, and Climate Change, located approximately 9 km from the hospital.

The study was carried out in accordance with the principles of the Declaration of Helsinki, after local ethics committee approval and institutional permissions had been obtained.

### Patient selection and inclusion criteria

Patients aged 18 years and older who presented to the ED between April 2022 and April 2023 were retrospectively screened. To minimize the risk of missing potential COPD exacerbation cases, screening was not restricted solely to COPD diagnostic codes; an extended ICD-10 screening set was used through the hospital information system records, including codes for COPD (J44, J44.0, J44.1, J44.8), dyspnea (R06.0), cough (R05), fever (R50.9), pneumonia (J18.0, J18.1, J18.8, J18.9), and respiratory failure (R96.0, R96.9).

In a second step, ED notes, previous ED admissions, outpatient records, and medical history of all candidate cases were examined in detail to confirm the established diagnosis of COPD.

The diagnosis of COPD exacerbation was made according to the GOLD 2026 criteria: new onset or worsening of dyspnea and/or cough within the previous 14 days, increased sputum production, prolonged expiratory phase and/or rhonchi on physical examination, and the need for bronchodilator and/or systemic corticosteroid treatment. Alternative diagnoses that may mimic COPD exacerbation, such as heart failure, pulmonary embolism, or pneumothorax, were also evaluated (Global Initiative for Chronic Obstructive Lung Disease, 2026).

### Exclusion criteria

Patients were excluded if any of the following applied:

- Inaccessible medical records.
- Absence of a complete blood count at admission.
- Residence outside the study region (to minimize exposure misclassification).
- Presence of comorbid conditions that could substantially affect hemogram parameters (sepsis, active malignancy, chemotherapy, granulocyte colony-stimulating factor use, or hematologic-oncologic disease).
- Phenotype meeting neither eosinophilic nor neutrophilic criteria (eosinophils < 200 cells/µL and < 2%, neutrophils < 65%, leukocyte count < 11,000/µL).
- Repeat admission within 30 days of a previous admission.

### Exacerbation phenotyping

Based on peripheral complete blood count results obtained at admission, exacerbations were classified into three groups according to previously published hematological phenotyping criteria (Kandemir et al. 2021; Kang et al. 2016; Bélanger et al. 2018; Şancı et al. 2023):

- Eosinophilic exacerbation: eosinophils > 200 cells/µL or > 2%.
- Neutrophilic exacerbation: neutrophil percentage > 65% or leukocyte count > 11,000/µL.
- Mixed-type exacerbation: simultaneous fulfillment of both criteria.

### Environmental data

Daily mean concentrations of air pollutants (PM₁₀, PM₂.₅, SO₂, NO₂, CO, and O₃) were calculated from hourly measurements obtained from the nearest air quality monitoring station of the Ministry of Environment, Urbanization, and Climate Change. Meteorological parameters – daily mean temperature (°C), relative humidity (%), atmospheric pressure (hPa), wind speed (m/s), and total precipitation (mm) – were obtained from the records of the Turkish State Meteorological Service.

Because the proportion of missing data was below 3% for all environmental variables, complete-case analysis was preferred and no missing data imputation was applied. Measured concentrations were compared with the World Health Organization (WHO) 2021 Air Quality Guidelines (AQG) for particulate matter (PM₂.₅ and PM₁₀), ozone (O₃), nitrogen dioxide (NO₂), sulfur dioxide (SO₂), and carbon monoxide (CO); this comparison was based on AQG levels rather than interim targets (World Health Organization 2021). Descriptive statistics and the number of days exceeding WHO thresholds are presented in Supplementary Table 1.

### Day-level outcome definitions

For each calendar day during the study period, the daily count of COPD exacerbation visits was calculated. In phenotype-specific analyses, daily counts of eosinophilic, neutrophilic, and mixed-type exacerbations were determined separately. Thus, when multiple visits occurred on the same calendar day or visits from different phenotypes coincided, all events were included in the analyses.

### Statistical analysis

Patient-level clinical analyses were performed with IBM SPSS Statistics 29.0 (IBM Corp., Armonk, NY, USA). Normality was assessed by the Kolmogorov–Smirnov and Shapiro–Wilk tests. Continuous variables are presented as mean ± standard deviation or median (IQR) according to distribution, and categorical variables as count and percentage. Between-group comparisons were performed using one-way ANOVA (Tukey post hoc), Kruskal–Wallis (Dunn post hoc), and Bonferroni-corrected chi-square tests. These analyses were descriptive and were not used for causal inference.

Day-level environmental analyses were performed with R 4.3.0 (packages dlnm, mgcv, survival, dplyr, and ggplot2). The primary outcome variable was the daily count of COPD exacerbation visits; in phenotype-specific analyses, daily counts for each hematological phenotype were modeled separately. Because of the overdispersion observed in the daily counts, a quasi-Poisson distribution and log link function were used.

Associations between environmental exposures and daily exacerbation counts were evaluated using single-pollutant distributed lag non-linear models (DLNM) fitted separately for each environmental parameter and each phenotype (Gasparrini 2011). The exposure–response relationship was modeled with a linear term, and the lag structure was modeled with a natural cubic spline (2 degrees of freedom; lag 0–3 days). Long-term time trend and seasonality were controlled with a natural cubic spline of 7 degrees of freedom per year; day of the week was included as a categorical covariate.

To minimize potential collinearity, all pollutants were analyzed using single-pollutant models. Correlations among environmental variables were examined using Pearson coefficients, and multicollinearity was assessed using variance inflation factors (VIF). Results are reported as relative risks (RR) per interquartile range (IQR) increase, with 95% confidence intervals (CI), for each lag day and for the cumulative lag 0–3 effect.

As a sensitivity analysis, a time-stratified case-crossover design was applied. In this approach, each exacerbation day was matched to control days falling on the same day of the week within the same calendar month; thus, seasonality, long-term trend, and day-of-week effects were structurally controlled. Associations were estimated using conditional logistic regression and reported as odds ratios (OR) with 95% CIs. Lag-specific case-crossover odds ratios for all environmental parameters are presented in Supplementary Tables 2A  and 2B.

Because DLNM models simultaneously estimate lag effects in a single smooth-spline framework rather than as independent tests at each lag, no classical multiple-comparison correction (Bonferroni/FDR) was applied (Gasparrini 2011). The robustness of findings was instead evaluated through an independent case-crossover sensitivity analysis, with priority given in interpretation to associations consistent across both methods. The analytical plan was primarily oriented toward exploration of environmental patterns and estimation of effect sizes. A two-sided *p* < 0.05 was considered statistically significant; results were interpreted together with effect estimates and confidence intervals. Detailed DLNM model specifications and collinearity diagnostics are provided in Supplementary Tables 3 A and 3B.

## Results

### Patient characteristics

**Fig. 1:**
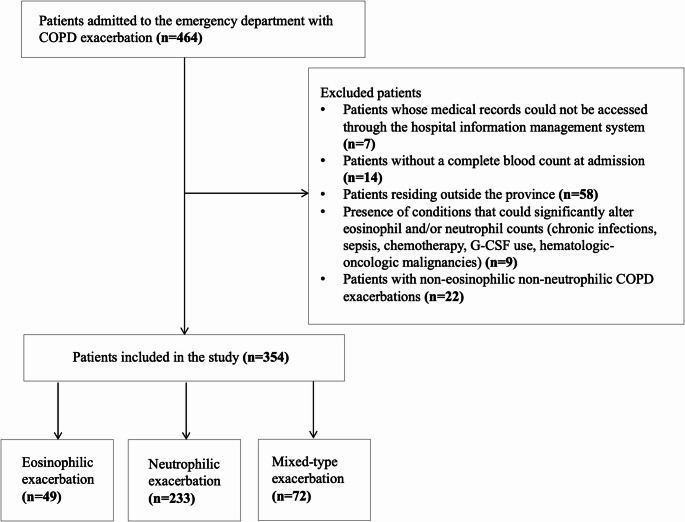

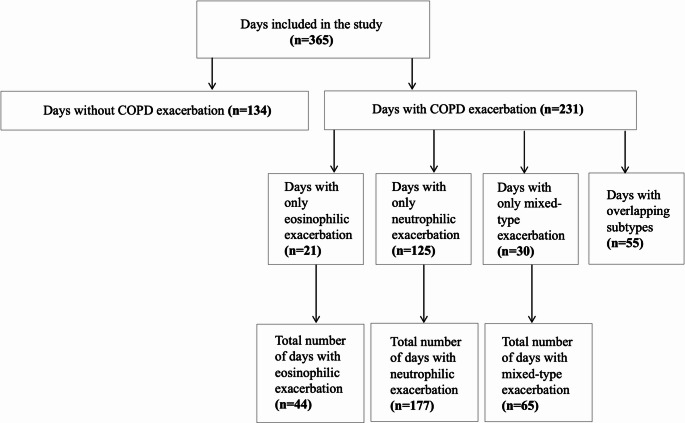
(**A**) Patient flow diagram. Of 464 patients admitted to the emergency department with COPD exacerbation between April 2022 and April 2023, 354 patients met the inclusion criteria and were stratified into eosinophilic (*n* = 49), neutrophilic (*n* = 233), and mixed-type (*n* = 72) exacerbation phenotypes. (**B**) Day-level distribution of the study period (*n* = 365 days), showing days without exacerbation (*n* = 134) and days with exacerbation (*n* = 231), further classified by phenotype

During the study period, 464 patients presented to the ED with COPD exacerbation. After applying the exclusion criteria, 354 patients were included in the analysis (Fig. 1A). Of the included patients, 68.1% were male and the mean age was 70 ± 10 years. According to hematological phenotype distribution, 233 patients (65.8%) had neutrophilic, 72 (20.3%) had mixed-type, and 49 (13.8%) had eosinophilic exacerbations (Table 1).

**Table 1:** Baseline characteristics of the study population

	All patients (*n* = 354)	Eosinophilic exacerbation (*n* = 49)	Neutrophilic exacerbation (*n* = 233)	Mixed-type exacerbation (*n* = 72)	*p* value
Male sex, n (%)	241. (68.1)	25 (51.0)	170 (73.0)	46. (63.9)	0.008^b^
Age, mean ± SD	70 ± 10	69 ± 11	70 ± 10	70 ± 10	0.735ᵃ
Hypertension, n (%)	194 (54.8)	28 (57.1)	129 (55.4)	37 (51.4)	0.788ᵇ
Diabetes mellitus, n (%)	98 (27.7)	15 (30.6)	64 (27.5)	19 (26.4)	0.892ᵇ
Coronary artery disease, n (%)	95 (26.8)	14 (28.6)	67 (28.8)	14 (19.4)	0.297ᵇ
Heart failure, n (%)	67 (18.9)	4 (8.2)	48 (20.6)	15 (20.8)	0.116ᵇ
Atrial fibrillation, n (%)	55 (15.5)	5 (10.2)	37 (15.9)	13 (18.1)	0.474ᵇ
Coronary artery bypass graft, n (%)	20 (5.6)	2 (4.1)	18 (7.7)	0 (0.0)	0.037ᵇ
Chronic kidney disease, n (%)	19 (5.4)	0 (0.0)	13 (5.6)	6 (8.3)	0.111ᵇ
Cerebrovascular event, n (%)	9 (2.5)	1 (2.0)	6 (2.6)	2 (2.8)	1.000ᵇ
Home oxygen use, n (%)	130 (36.7)	11 (22.4)	91 (39.1)	28 (38.9)	0.084ᵇ
Home BiPAP use, n (%)	35 (9.9)	2 (4.1)	26 (11.2)	7 (9.7)	0.329ᵇ
Radiological infiltration, n (%)	193 (54.5)	16 (32.7)	139 (59.7)	38 (52.8)	0.003ᵇ
ICU admission, n (%)	35 (9.9)	2 (4.1)	30 (12.9)	3 (4.2)	0.039ᵇ
30-day mortality, n (%)	43 (12.1)	1 (2.0)	35 (15.0)	7 (9.7)	0.028ᵇ
Vital Signs					
Body temperature (°C), median (IQR)	36.7 (36.4–36.9)	36.6 (36.4–36.8)	36.7 (36.4–36.9)	36.7 (36.5–36.9)	0.128ᶜ
Heart rate (bpm), mean ± SD	103 ± 21.2	89 ± 15.2	106 ± 21.3	102 ± 20.8	< 0.001ᵃ
Respiratory rate, median (IQR)	28 (26–34)	28 (24–32)	30 (28–36)	28 (26–32)	0.005ᶜ
Systolic BP (mmHg), median (IQR)	138 (125–154)	140 (129–154)	137 (123–155)	138 (126–150)	0.533ᶜ
Diastolic BP (mmHg), median (IQR)	77 (69–86)	79 (65–86)	77 (70–87)	76 (68–86)	0.628ᶜ
Oxygen saturation (%), median (IQR)	92 (85–95)	94 (90–97)	90 (83–95)	92 (85–95)	< 0.001ᶜ
Laboratory Parameters					
pH, median (IQR)	7.40 (7.36–7.44)	7.38 (7.36–7.41)	7.40 (7.35–7.44)	7.39 (7.38–7.44)	0.029ᶜ
pCO₂ (mmHg), median (IQR)	42.3 (36.0–50.5)	42.6 (40.6–50.5)	41.9 (35.8–50.9)	44.0 (34.3–49.3)	0.337ᶜ
Lactate (mg/dL), median (IQR)	15 (11–20)	14 (11–19)	16 (12–22)	14 (10–18)	0.017ᶜ
CRP (mg/L), median (IQR)	35 (9–103)	6 (2–18)	51 (15–112)	42 (12–99)	< 0.001ᶜ

Abbreviations: *BP* blood pressure, *BiPAP* bilevel positive airway pressure, *CRP* C-reactive protein, *ICU* intensive care unit, IQR interquartile range (25th–75th percentile), pCO_2_, partial pressure of carbon dioxide, *SD* standard deviation

Statistical methods: ᵃ One-way analysis of variance (Tukey post hoc test). ᵇ Chi-square test (Bonferroni correction). ᶜ Kruskal–Wallis test (Dunn post hoc test)

Significant differences in clinical severity were observed across phenotypes. The neutrophilic phenotype was associated with lower oxygen saturation, higher CRP and leukocyte counts, more frequent radiological infiltration, higher rates of intensive care unit admission, and higher 30-day mortality. The eosinophilic phenotype showed the mildest clinical presentation, while the mixed-type phenotype generally exhibited intermediate features between the two extremes (Table 1).

### Temporal and seasonal distribution

Of the 365 days during the study period, at least one COPD exacerbation visit was recorded on 231 days (63.3%); 134 days had no exacerbation visits (Fig. 1B).

**Fig. 2:**
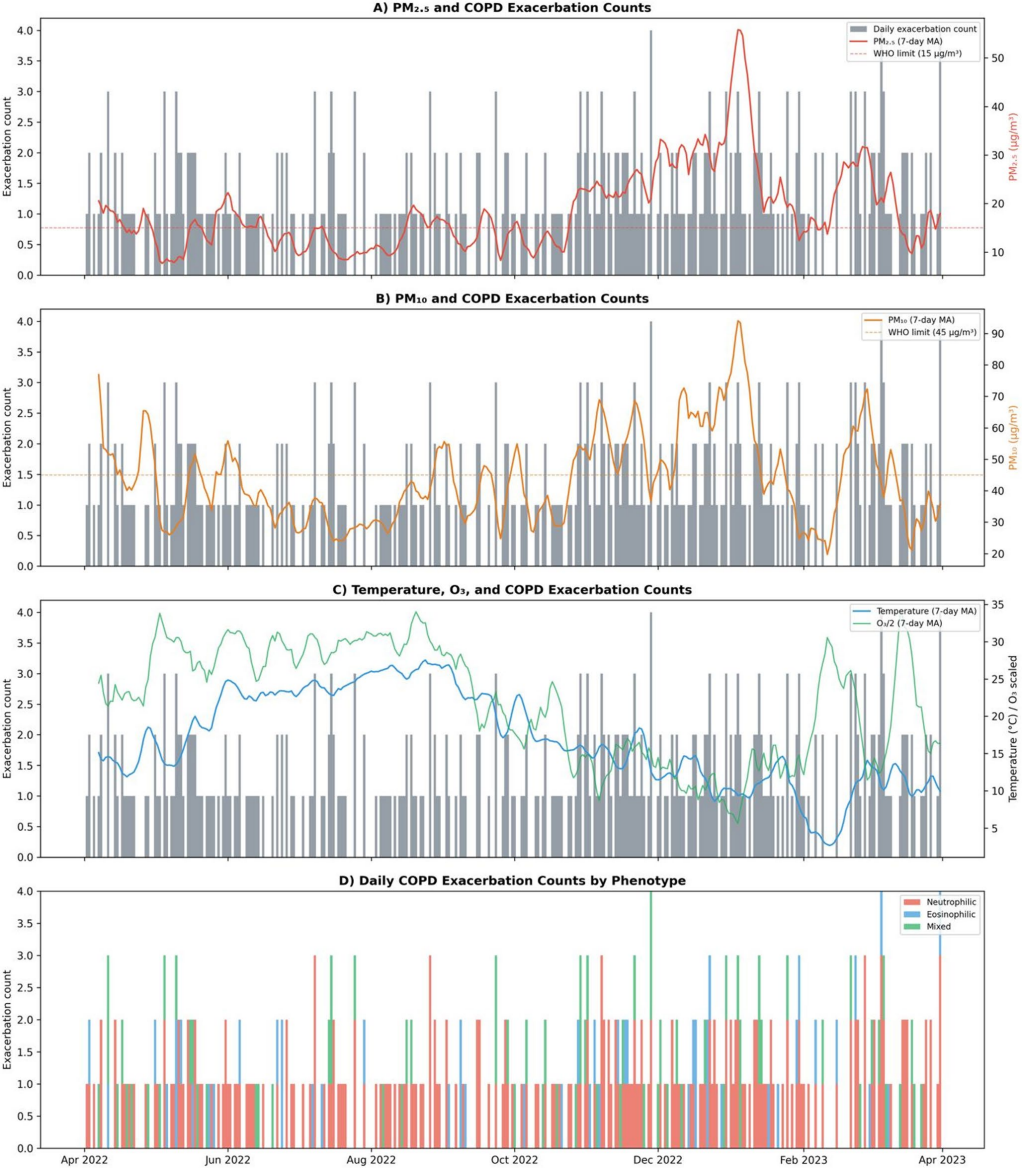
Time series of daily COPD exacerbation counts (gray bars) and selected environmental parameters (7-day moving average, colored lines) over the study period (April 2022–April 2023). (**A**) PM₂.₅ concentrations with the WHO 24-hour guideline level (15 µg/m³) shown as a dashed line. (**B**) PM₁₀ concentrations with the WHO 24-hour guideline level (45 µg/m³) shown as a dashed line. (**C**) Daily mean temperature and O₃ concentrations. (**D**) Daily COPD exacerbation counts colored by hematological phenotype

COPD exacerbations were more frequent during winter and early spring and less frequent during summer. PM₂.₅ levels increased during winter months and exceeded the WHO 24-hour guideline level on a substantial number of days throughout the year. O₃ levels peaked during summer. The temporal distribution of environmental parameters during the study period is shown in Fig. 2 (selected parameters) and Supplementary Fig. 1 (all parameters); descriptive statistics are presented in Supplementary Table 1.

### Associations between environmental parameters and COPD exacerbation counts: distributed lag non-linear model (DLNM)

**Fig. 3:**
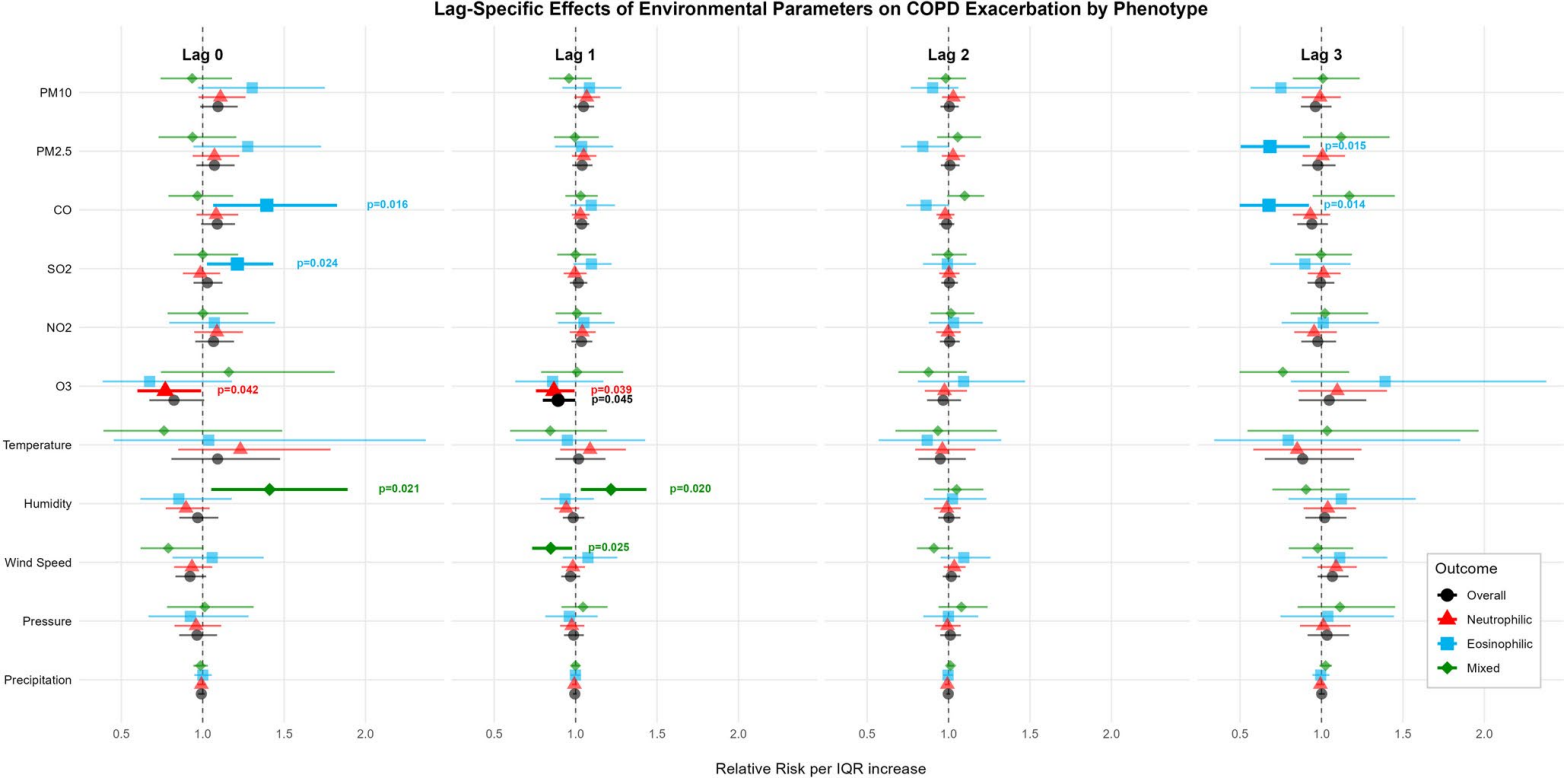
Lag-specific effects of environmental parameters on COPD exacerbation counts by hematological phenotype, derived from quasi-Poisson distributed lag non-linear models (DLNM). Each panel shows the relative risk (RR) per interquartile range (IQR) increase with 95% confidence intervals at lag 0, 1, 2, and 3. Statistically significant associations (*p* < 0.05) are highlighted with annotated p values. Air pollutants (PM₁₀, PM₂.₅, CO, SO₂, NO₂, O₃) and meteorological parameters (temperature, humidity, wind speed, pressure, precipitation) are shown for the overall cohort and three phenotypes

**Fig. 4:**
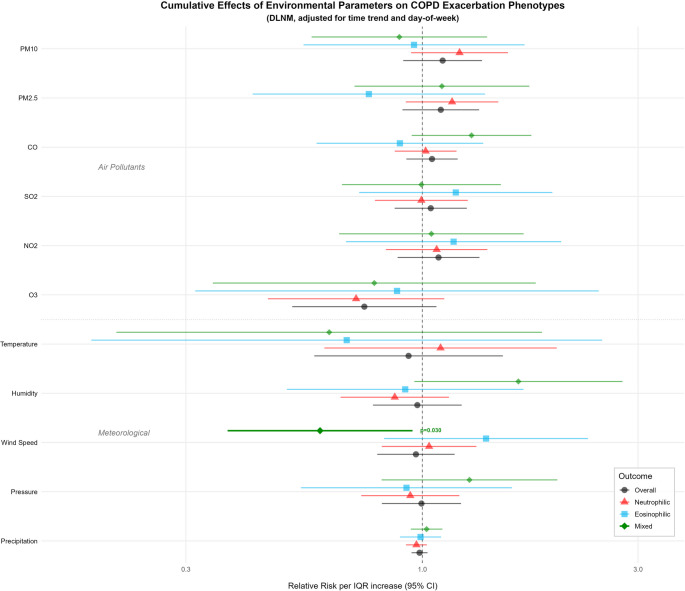
Cumulative effects (lag 0–3) of environmental parameters on COPD exacerbation counts by hematological phenotype, derived from quasi-Poisson distributed lag non-linear models (DLNM) adjusted for time trend and day-of-week. The relative risk (RR) per IQR increase is shown with 95% confidence intervals for the overall cohort and three phenotypes. Statistically significant associations (*p* < 0.05) are highlighted with annotated p values

In the quasi-Poisson DLNM analysis, associations between environmental exposures and daily COPD exacerbation counts varied by phenotype (Tables 2 and 3; Figs. 3 and 4).

**Table 2:** A. Associations of air pollutants with daily COPD exacerbation counts: quasi-poisson distributed lag non-linear model (DLNM) results by hematological phenotype

Parameter (IQR)	Lag	Eosinophilic RR (95% CI)	Neutrophilic RR (95% CI)	Mixed-type RR (95% CI)	Overall COPD RR (95% CI)
PM₁₀ (IQR: 26.9 µg/m³)	lag 0	1.30 (0.97–1.75)	1.11 (0.97–1.26)	0.94 (0.74–1.18)	1.09 (0.98–1.22)
	lag 1	1.08 (0.92–1.28)	1.07 (0.99–1.15)	0.96 (0.84–1.10)	1.05 (0.99–1.11)
	lag 2	0.90 (0.77–1.06)	1.03 (0.96–1.10)	0.98 (0.87–1.11)	1.00 (0.95–1.06)
	lag 3	0.75 (0.56–1.00)	0.99 (0.88–1.12)	1.01 (0.82–1.23)	0.96 (0.87–1.06)
	Cumulative	0.96 (0.55–1.68)	1.21 (0.94–1.55)	0.89 (0.57–1.39)	1.11 (0.91–1.36)
PM₂.₅ (IQR: 12.7 µg/m³)	lag 0	1.28 (0.94–1.73)	1.07 (0.94–1.23)	0.94 (0.73–1.21)	1.07 (0.96–1.20)
	lag 1	1.04 (0.87–1.23)	1.05 (0.98–1.13)	1.00 (0.87–1.14)	1.04 (0.98–1.10)
	lag 2	0.84 (0.71–1.00)	1.03 (0.96–1.10)	1.06 (0.93–1.20)	1.01 (0.95–1.07)
	lag 3	0.68* (0.50–0.93)	1.01 (0.88–1.14)	1.12 (0.89–1.42)	0.98 (0.88–1.09)
	Cumulative	0.76 (0.42–1.38)	1.16 (0.92–1.47)	1.10 (0.71–1.72)	1.10 (0.90–1.34)
CO (IQR: 398.3 µg/m³)	lag 0	1.39* (1.06–1.82)	1.08 (0.96–1.22)	0.97 (0.79–1.19)	1.09 (0.99–1.20)
	lag 1	1.10 (0.97–1.24)	1.03 (0.98–1.09)	1.03 (0.94–1.14)	1.04 (0.99–1.08)
	lag 2	0.86 (0.74–1.00)	0.98 (0.93–1.04)	1.10 (0.99–1.22)	0.99 (0.94–1.03)
	lag 3	0.68* (0.50–0.92)	0.93 (0.82–1.05)	1.17 (0.95–1.45)	0.94 (0.85–1.04)
	Cumulative	0.89 (0.58–1.36)	1.02 (0.87–1.19)	1.28 (0.95–1.74)	1.05 (0.92–1.20)
SO₂ (IQR: 1.2 µg/m³)	lag 0	1.21* (1.03–1.43)	0.99 (0.88–1.11)	1.00 (0.82–1.22)	1.03 (0.94–1.12)
	lag 1	1.10 (0.99–1.22)	0.99 (0.93–1.07)	1.00 (0.89–1.13)	1.02 (0.96–1.07)
	lag 2	0.99 (0.84–1.17)	1.00 (0.94–1.07)	1.00 (0.90–1.11)	1.00 (0.96–1.06)
	lag 3	0.90 (0.68–1.18)	1.01 (0.92–1.12)	1.00 (0.84–1.19)	0.99 (0.91–1.08)
	Cumulative	1.19 (0.73–1.94)	1.00 (0.79–1.26)	1.00 (0.66–1.49)	1.04 (0.87–1.25)
NO₂ (IQR: 7.6 µg/m³)	lag 0	1.07 (0.79–1.44)	1.09 (0.95–1.25)	1.00 (0.78–1.28)	1.07 (0.95–1.19)
	lag 1	1.05 (0.89–1.24)	1.04 (0.96–1.12)	1.01 (0.88–1.16)	1.04 (0.97–1.10)
	lag 2	1.03 (0.88–1.21)	1.00 (0.92–1.08)	1.01 (0.89–1.16)	1.01 (0.95–1.07)
	lag 3	1.01 (0.76–1.35)	0.95 (0.83–1.09)	1.02 (0.81–1.29)	0.98 (0.88–1.09)
	Cumulative	1.17 (0.68–2.03)	1.08 (0.83–1.39)	1.05 (0.65–1.68)	1.09 (0.88–1.34)
O₃ (IQR: 29.7 µg/m³)	lag 0	0.67 (0.39–1.18)	0.77* (0.60–0.99)	1.16 (0.74–1.81)	0.82 (0.67–1.01)
	lag 1	0.86 (0.63–1.17)	0.87* (0.76–0.99)	1.01 (0.79–1.29)	0.89* (0.80–1.00)
	lag 2	1.09 (0.81–1.47)	0.97 (0.85–1.11)	0.88 (0.69–1.11)	0.97 (0.87–1.08)
	lag 3	1.39 (0.81–2.38)	1.10 (0.86–1.40)	0.76 (0.50–1.17)	1.05 (0.86–1.28)
	Cumulative	0.88 (0.31–2.46)	0.71 (0.46–1.12)	0.78 (0.34–1.78)	0.74 (0.52–1.07)

Abbreviations: *RR* relative risk; 95% CI, 95% confidence interval, *IQR* interquartile range, *DLNM* distributed lag non-linear model, *COPD* chronic obstructive pulmonary disease, *PM* particulate matter, *CO* carbon monoxide, *SO*₂ sulfur dioxide, *NO*₂ nitrogen dioxide, *O*₃ ozone. Model: Quasi-Poisson regression with a log link function. Exposure–response modeled with a linear term and lag structure with a natural cubic spline (2 df; lag 0–3 days). Long-term time trend and seasonality controlled with a natural cubic spline of 7 df per year, day of the week included as a categorical covariate. Each pollutant analyzed in a separate single-pollutant model

Results: Results presented as relative risks (RR) with 95% CIs per IQR increase of the corresponding parameter. The “Cumulative” row indicates the combined effect across lag 0–3. * p < 0.05; statistically significant results are shown in bold with an asterisk (*)

**Table 3:** Associations of meteorological parameters with daily COPD exacerbation counts: quasi-Poisson distributed lag non-linear model (DLNM) results by hematological phenotype.

Parameter (IQR)	Lag	Eosinophilic RR(95% CI)	Neutrophilic RR(95% CI)	Mixed-type RR(95% CI)	Overall COPD RR(95% CI)
Temperature (IQR: 11.3 °C)	lag 0	1.04 (0.45–2.37)	1.23 (0.85–1.78)	0.76 (0.39–1.49)	1.09 (0.81–1.48)
	lag 1	0.95 (0.63–1.43)	1.09 (0.91–1.31)	0.84 (0.60–1.19)	1.02 (0.88–1.18)
	lag 2	0.87 (0.57–1.32)	0.96 (0.80–1.16)	0.93 (0.67–1.30)	0.95 (0.81–1.11)
	lag 3	0.80 (0.34–1.85)	0.85 (0.58–1.25)	1.03 (0.55–1.96)	0.88 (0.65–1.20)
	Cumulative	0.68 (0.19–2.50)	1.10 (0.61–1.98)	0.62 (0.21–1.84)	0.93 (0.58–1.51)
Humidity (IQR: 20.4%)	lag 0	0.85 (0.62–1.18)	0.90 (0.77–1.04)	1.41* (1.05–1.89)	0.97 (0.86–1.10)
	lag 1	0.93 (0.79–1.11)	0.94 (0.87–1.02)	1.22* (1.03–1.43)	0.99 (0.92–1.05)
	lag 2	1.02 (0.85–1.23)	0.99 (0.91–1.08)	1.05 (0.91–1.21)	1.00 (0.94–1.07)
	lag 3	1.12 (0.80–1.58)	1.04 (0.89–1.21)	0.91 (0.70–1.17)	1.02 (0.90–1.15)
	Cumulative	0.92 (0.50–1.67)	0.87 (0.66–1.15)	1.63 (0.96–2.77)	0.98 (0.78–1.22)
Wind speed (IQR: 0.5 m/s)	lag 0	1.06 (0.81–1.37)	0.93 (0.82–1.06)	0.79 (0.62–1.01)	0.92 (0.83–1.02)
	lag 1	1.08 (0.92–1.26)	0.98 (0.91–1.06)	0.85* (0.73–0.98)	0.97 (0.91–1.03)
	lag 2	1.09 (0.95–1.26)	1.03 (0.97–1.10)	0.91 (0.81–1.03)	1.02 (0.96–1.07)
	lag 3	1.11 (0.88–1.40)	1.09 (0.98–1.22)	0.98 (0.80–1.20)	1.07 (0.98–1.17)
	Cumulative	1.38 (0.82–2.32)	1.04 (0.81–1.32)	0.59* (0.37–0.95)	0.97 (0.80–1.18)
Atmospheric pressure (IQR: 8.4 hPa)	lag 0	0.92 (0.67–1.28)	0.96 (0.83–1.11)	1.01 (0.78–1.31)	0.96 (0.86–1.09)
	lag 1	0.96 (0.81–1.14)	0.98 (0.90–1.05)	1.05 (0.91–1.20)	0.99 (0.93–1.05)
	lag 2	1.00 (0.84–1.18)	0.99 (0.92–1.07)	1.08 (0.94–1.24)	1.01 (0.95–1.08)
	lag 3	1.04 (0.75–1.45)	1.01 (0.87–1.18)	1.11 (0.85–1.45)	1.03 (0.91–1.17)
	Cumulative	0.92 (0.54–1.58)	0.94 (0.73–1.21)	1.27 (0.81–1.99)	1.00 (0.81–1.22)
Precipitation (IQR: 1.0 mm)	lag 0	1.00 (0.95–1.06)	0.99 (0.97–1.02)	0.99 (0.94–1.03)	0.99 (0.97–1.01)
	lag 1	1.00 (0.97–1.03)	0.99 (0.98–1.01)	1.00 (0.97–1.02)	0.99 (0.98–1.01)
	lag 2	1.00 (0.97–1.03)	0.99 (0.98–1.01)	1.01 (0.99–1.03)	1.00 (0.99–1.01)
	lag 3	1.00 (0.94–1.05)	0.99 (0.97–1.02)	1.03 (0.99–1.06)	1.00 (0.98–1.02)
	Cumulative	0.99 (0.89–1.10)	0.97 (0.92–1.02)	1.02 (0.94–1.11)	0.99 (0.95–1.03)

Abbreviations: RR, relative risk; 95% CI, 95% confidence interval; IQR, interquartile range; DLNM, distributed lag non-linear model; COPD, chronic obstructive pulmonary disease; °C, degrees Celsius; hPa, hectopascal; m/s, meters per second; mm, millimeters. Model: Quasi-Poisson regression with a log link function. Exposure–response modeled with a linear term and lag structure with a natural cubic spline (2 df; lag 0–3 days). Long-term time trend and seasonality controlled with a natural cubic spline of 7 df per year; day of the week included as a categorical covariate. Each meteorological parameter analyzed in a separate model

Results:Results presented as relative risks (RR) with 95% CIs per IQR increase of the corresponding parameter. The “Cumulative” row indicates the combined effect across lag 0–3.*p < 0.05; statistically significant results are shown in bold with an asterisk (*)

No significant cumulative association was identified for the overall COPD exacerbation count for any of the air pollutants or meteorological parameters examined (Tables 02 and 3B; Fig. 4).

#### Eosinophilic phenotype

A positive association was observed between eosinophilic exacerbation count and CO at lag 0 (RR = 1.39; 95% CI: 1.06–1.82; *p* = 0.016). SO₂ also showed a significant positive association at lag 0 (RR = 1.21; 95% CI: 1.03–1.43; *p* = 0.024). At lag 3, inverse associations were observed for both PM₂.₅ (RR = 0.68; 95% CI: 0.50–0.93; *p* = 0.015) and CO (RR = 0.68; 95% CI: 0.50–0.92; *p* = 0.014). Cumulative effects did not reach statistical significance (Table 2A; Figs. 3 and 4).

#### Neutrophilic phenotype

Inverse associations between neutrophilic exacerbation count and O₃ were observed at lag 0 (RR = 0.77; 95% CI: 0.60–0.99; *p* = 0.042) and lag 1 (RR = 0.87; 95% CI: 0.76–0.99; *p* = 0.039); however, these findings were not confirmed in the case-crossover sensitivity analysis. No significant associations were observed for other pollutants or meteorological parameters (Table 2A; Fig. 3).

#### Mixed-type phenotype

Positive associations between mixed-type exacerbation count and humidity were observed at lag 0 (RR = 1.41; 95% CI: 1.05–1.89; *p* = 0.021) and lag 1 (RR = 1.22; 95% CI: 1.03–1.43; *p* = 0.020). Wind speed was associated with lower exacerbation counts at lag 1 (RR = 0.85; 95% CI: 0.73–0.98; *p* = 0.025), and the cumulative effect of wind speed was also significant (RR = 0.59; 95% CI: 0.37–0.95; *p* = 0.030). No significant associations were observed for air pollutants (Table 2B; Figs. 3 and 4).

### Sensitivity analysis: case-crossover design

In the time-stratified case-crossover analysis, several DLNM findings were confirmed and phenotype-specific associations were supported (Supplementary Tables 2 A and 2B; Supplementary Figs. 2 and 3).

In the eosinophilic phenotype, a significant association with SO₂ was observed at lag 1 (OR = 1.43; 95% CI: 1.03–2.00; *p* = 0.034) (Supplementary Table 2 A; Supplementary Fig. 3).

In the mixed-type phenotype, the positive association with humidity persisted at lag 0 (OR = 1.69; 95% CI: 1.04–2.74; *p* = 0.033), lag 1 (OR = 1.86; 95% CI: 1.09–3.19; *p* = 0.023), and at the cumulative level (OR = 1.73; 95% CI: 1.01–2.97; *p* = 0.046) (Supplementary Table 2B; Supplementary Figs. 2 and 3).

Wind speed showed an inverse association in the mixed-type phenotype at lag 1 (OR = 0.49; 95% CI: 0.30–0.81; *p* = 0.005) and at the cumulative level (OR = 0.65; 95% CI: 0.43–0.98; *p* = 0.039) (Supplementary Table 2B; Supplementary Figs. 2 and 3).

An association with atmospheric pressure was also observed at lag 1 in the mixed-type phenotype (OR = 1.73; 95% CI: 1.03–2.92; *p* = 0.040) (Supplementary Table 2B; Supplementary Fig. 3); however, this finding was limited to a single lag and is therefore reported with caution.

No significant associations were identified between any environmental parameter and the neutrophilic phenotype in the case-crossover analysis (Supplementary Tables 2 A and 2B).

## Discussion

In this study, we evaluated the associations between short-term environmental exposures and COPD exacerbations classified by hematological phenotype. The main findings can be summarized in three points. First, no independent significant association was identified between air pollutants or meteorological parameters and the overall COPD exacerbation count. Second, in phenotype-specific analyses, eosinophilic exacerbations showed significant associations with short-term SO₂ and CO exposure. Third, mixed-type exacerbations displayed consistent associations with high humidity and low wind speed, whereas no clear environmental associations were identified in the neutrophilic phenotype after temporal adjustment. These findings suggest that COPD exacerbations are not a homogeneous clinical event with respect to environmental triggers, and that phenotype-based approaches may be more informative for explaining environmental risks.

### Clinical severity and prognostic features of hematological phenotypes

In our study, marked clinical differences were observed across hematological phenotypes. The neutrophilic phenotype represented the most severe clinical presentation, with lower oxygen saturation, higher inflammatory markers, more frequent radiological infiltration, higher rates of intensive care unit admission, and higher 30-day mortality. In contrast, the eosinophilic phenotype showed a milder course, while the mixed-type phenotype generally exhibited an intermediate profile.

Kandemir et al. similarly reported neutrophilic exacerbations as more severe and eosinophilic exacerbations as relatively benign in clinical course (Kandemir et al. 2021). Other studies have also reported this consistent pattern (Bélanger et al. 2018; Kang et al. 2016). Thus, hematological phenotyping may serve as a practical tool for early risk stratification and clinical decision-making in the ED.

### Limited environmental associations in the overall COPD exacerbation population

The effect of short-term air pollution exposure on COPD exacerbations has been extensively investigated; various pollutants have been reported to be associated with COPD-related ED visits and hospitalizations (Li et al. 2016; Liu et al. 2022; Javorac et al. 2021). However, the magnitude and direction of these associations vary considerably depending on geographic region, pollutant profile, climate, the phenotypic composition of the patient population, and the statistical methodology used.

In our study, when all COPD exacerbations were evaluated without phenotype stratification, no significant association was identified after temporal adjustment. We propose two complementary explanations. First, in our study region, particulate matter levels rise markedly during winter, when COPD exacerbations also increase; O₃, in contrast, follows the opposite seasonal pattern, peaking in summer. These coinciding seasonal patterns create a setting prone to strong seasonal confounding (Tseng et al. 2013). Second, given that eosinophilic and neutrophilic phenotypes appear to respond to different environmental triggers, analyzing these subgroups as a single population may have led to attenuation of phenotype-specific effects in pooled analyses. Indeed, when phenotype-specific analyses were performed, environmental patterns not visible in the overall analysis became more discernible.

### Eosinophilic phenotype: short-lag associations with gaseous pollutants

The significant positive associations of SO₂ and CO at lag 0 in eosinophilic exacerbations suggest that this phenotype may exhibit greater short-term susceptibility to gaseous pollutants. The fact that both pollutants reached significance on the same day is consistent with biological hypotheses suggesting that type-2 inflammation–dominated airway responses may react more rapidly to environmental triggers (David et al. 2021; Narendra and Hanania 2019). It has also been reported in the literature that respiratory effects of air pollution exposure may be more pronounced in individuals with higher blood eosinophil counts (Nurhussien et al. 2022).

The inverse associations observed at lag 3 for both PM₂.₅ and CO may relate to the so-called “harvesting effect”, whereby susceptible individuals present earlier following high-exposure days, resulting in a transient reduction in subsequent event counts. The persistence of a significant association for SO₂ at lag 1 in the sensitivity analysis (OR = 1.43) suggests some degree of consistency in this finding. These findings may help inform future targeted preventive strategies for type-2-dominant COPD phenotypes.

Nevertheless, given the limited number of eosinophilic exacerbation days (*n* = 44), estimates in this phenotype carry wide confidence intervals and should be interpreted with caution. The inverse lag 3 findings, in particular, should be considered hypothesis-generating and require confirmation in larger, multicenter studies.

### Neutrophilic phenotype: limited environmental associations after temporal adjustment

Although the neutrophilic phenotype represented the majority of COPD exacerbations in our cohort (65.8%), it is notable that no environmental parameter showed an independent significant association with this phenotype after temporal adjustment. The inverse association with O₃ observed in the DLNM analysis was not confirmed in the case-crossover sensitivity analysis, suggesting that this finding likely reflects residual seasonal confounding rather than a causal protective effect. As O₃ peaks in summer while neutrophilic exacerbations cluster in winter, strong seasonal correlations with temperature and other pollutants may not have been fully modeled. The inverse O₃ association is therefore not interpreted as a biological protective effect.

One possible explanation for this pattern is that neutrophilic exacerbations may be driven more by infectious triggers than by short-term environmental fluctuations. Neutrophil-dominant COPD exacerbations have previously been linked more frequently to bacterial and viral infections (Doğan et al. 2021; Kandemir et al. 2021). In addition, the short lag 0–3 lag window may have been insufficient to capture longer-term environmental effects in this phenotype. Further studies incorporating longer lag windows and infection markers are needed for the neutrophilic phenotype.

### Mixed-type phenotype: consistent associations with meteorological parameters

In this study, the mixed-type phenotype was defined as exacerbations simultaneously meeting eosinophilic and neutrophilic criteria. This definition may capture a true subgroup with concurrent activation of type-2 and innate immune pathways, but it may also partially reflect systemic inflammatory responses or coincidental eosinophilia in the setting of infection. Sputum-based classification would likely define a more homogeneous subgroup. Nevertheless, in this study, mixed-type exacerbations showed consistent associations with meteorological parameters – particularly high humidity and low wind speed – independently of air pollutants. The persistence of this pattern across both DLNM and case-crossover sensitivity analyses supports the consistency of this finding.

The significant short-lag association of high humidity suggests that the effect may emerge on the same day and the following day. Associations of humidity with COPD exacerbations have been previously reported in different populations; high humidity has been suggested to enhance the viability of respiratory pathogens and impair mucociliary clearance (Javorac et al. 2021; Ferrari et al. 2012). The protective association of wind speed at the cumulative level may be explained by enhanced atmospheric mixing leading to reduced pollutant accumulation. The persistence of humidity and wind-speed associations in the case-crossover analysis supports the relative consistency of meteorological signals in the mixed-type phenotype.

### Clinical and public health implications

The findings of this study suggest that phenotype-based environmental risk assessment in COPD exacerbations may be more informative than approaches based on the overall population. The phenotype-specific environmental patterns identified here suggest that surveillance strategies considering biological subgroups may merit further investigation. In particular, joint interpretation of air quality and meteorological data in heavily industrialized regions may help anticipate high-risk periods (GOLD 2026; Doğan et al. 2021).

These implications, however, are derived from a single-center observational study and should not be transferred directly into clinical practice without confirmatory evidence. To assess the impact of phenotype-based environmental surveillance on patient outcomes, larger, multicenter, prospective studies with longer follow-up and individual-level exposure assessment are needed.

### Limitations

This study has several methodological limitations. The single-center, retrospective design limits generalizability, and findings may be specific to an urban region with intensive industrial activity. Exposure was assigned at the population level using data from the nearest official regional monitoring station, located approximately 9 km from the hospital; while this approach reflects average population-level exposure, it cannot capture individual-level exposure differences, introduces a risk of exposure misclassification, and precludes individual-level causal inference.

Smoking status, pack-year history, and current smoking information were not consistently documented owing to retrospective record limitations and could therefore not be incorporated as individual-level covariates in the day-level environmental models; in the case-crossover design, however, time-invariant smoking history may be partially controlled, as each case serves as its own control.

Other individual-level confounders such as comorbidities, medication adherence, and infection status, as well as community-level activity of influenza and other seasonal respiratory viruses, COVID-19 waves, airborne pollen and fungal spores, and other environmental-biological variables, could not be controlled. Moreover, the study period (April 2022–April 2023) overlapped with the late phase of the COVID-19 pandemic, which may have influenced healthcare-seeking behavior or respiratory infection patterns.

Hematological phenotyping was based on peripheral blood counts. Sputum-based methods are biologically more specific but are not feasible for routine use in the ED setting. The reported correlation between peripheral blood eosinophils and sputum eosinophilia supports the clinical applicability of this approach (GOLD 2026; Doğan et al. 2021). The diagnosis of COPD relied on clinical records rather than spirometric confirmation, which does not exclude diagnostic uncertainty in some cases. The limited number of daily eosinophilic exacerbations (44 in total) restricted statistical power, and the short lag 0–3 window may have been insufficient to capture longer-term effects in some phenotypes.

Despite these limitations, the study has notable strengths. The phenotype-stratified design represents a methodological approach that is rarely applied in the environmental epidemiology of COPD exacerbations. Joint application of quasi-Poisson DLNM and case-crossover sensitivity analysis provided additional methodological assurance for associations consistent across both methods.

### Conclusion

This study suggests that COPD exacerbations are not a homogeneous clinical event with respect to environmental triggers. Whereas no significant environmental association was identified in the overall exacerbation population, selective environmental patterns emerged in subgroup analyses by hematological phenotype. Our findings suggest that phenotype-based environmental risk modeling may improve the identification of high-risk periods for COPD exacerbations in future studies.

These findings are hypothesis-generating; before translation into clinical practice, they require confirmation in larger, multicenter, prospective studies incorporating individual-level exposure assessment.

## Supplementary Information

Below is the link to the electronic supplementary material.

Supplementary Material 1: Daily COPD exacerbation counts (gray bars) plotted alongside the time series of all environmental parameters (red lines, smoothed values; blue dashed lines, WHO or reference thresholds where applicable) over the study period: PM₂.₅, PM₁₀, SO₂, NO₂, CO, O₃, temperature, precipitation, humidity, wind speed, and atmospheric pressure. The bottom panel shows the temporal distribution of daily exacerbation counts colored by hematological phenotype

Supplementary Material 2:Cumulative effects (lag 0–3) of environmental parameters on COPD exacerbation counts by hematological phenotype, derived from time-stratified case-crossover analyses. Odds ratios (OR) per IQR increase are shown with 95% confidence intervals for the overall cohort and three phenotypes. Statistically significant associations (p < 0.05) are highlighted with annotated p values

Supplementary Material 3: Lag-specific effects of environmental parameters on COPD exacerbation counts by hematological phenotype, derived from time-stratified case-crossover analyses. Each panel shows odds ratios (OR) per IQR increase with 95% confidence intervals at lag 0, 1, 2, and 3. Statistically significant associations (p < 0.05) are highlighted with annotated p values

Supplementary Material 4

## Data Availability

Research data are stored by the corresponding author and may be provided upon reasonable request.

## References

[CR1] Bélanger M, Couillard S, Courteau J, Larivée P, Poder TG, Carrier N, Girard K, Vézina FA, Vanasse A (2018) Eosinophil counts in first COPD hospitalizations: a comparison of health service utilization. Int J Chron Obstruct Pulmon Dis 13:3045–3054. 10.2147/COPD.S170743 30319252 10.2147/COPD.S170743 PMC6171756

[CR2] David B, Bafadhel M, Koenderman L, De Soyza A (2021) Eosinophilic inflammation in COPD: from an inflammatory marker to a treatable trait. Thorax 76:188–195. 10.1136/thoraxjnl-2020-215167 33122447 10.1136/thoraxjnl-2020-215167 PMC7815887

[CR3] Doğan NÖ, Varol Y, Köktürk N, Aksay E, Alpaydın AÖ, Çorbacıoğlu ŞK, Aksel G, Baha A, Akoğlu H, Karahan S, Şen E, Ergan B, Bayram B, Yılmaz S, Gürgün A, Polatlı M (2021) 2021 Guideline for the Management of COPD Exacerbations: Emergency Medicine Association of Turkey (EMAT) / Turkish Thoracic Society (TTS) Clinical Practice Guideline Task Force. Turk J Emerg Med 21:137–176. 10.4103/2452-2473.329630 34849428 10.4103/2452-2473.329630 PMC8593424

[CR4] Ferrari U, Exner T, Wanka ER, Bergemann C, Meyer-Arnek J, Hildenbrand B, Tufman A, Heumann C, Huber RM, Bittner M, Fischer R (2012) Influence of air pressure, humidity, solar radiation, temperature, and wind speed on ambulatory visits due to chronic obstructive pulmonary disease in Bavaria, Germany. Int J Biometeorol 56:137–143. 10.1007/s00484-011-0405-x 21301889 10.1007/s00484-011-0405-x

[CR5] Gasparrini A (2011) Distributed lag linear and non-linear models in R: the package dlnm. J Stat Softw 43:1–20. 10.18637/jss.v043.i08 PMC3191524 22003319

[CR6] Global Initiative for Chronic Obstructive Lung Disease (2026) 2026 GOLD Report: Global Strategy for Prevention, Diagnosis and Management of COPD. Available at: https://goldcopd.org/2026-gold-report/. Accessed April 1, 2026

[CR7] Hogea SP, Tudorache E, Fildan AP, Fira-Mladinescu O, Marc M, Oancea C (2020) Risk factors of chronic obstructive pulmonary disease exacerbations. Clin Respir J 14:183–197. 10.1111/crj.13129 31814260 10.1111/crj.13129

[CR8] Javorac J, Jevtić M, Živanović D, Ilić M, Bijelović S, Dragić N (2021) What are the effects of meteorological factors on exacerbations of chronic obstructive pulmonary disease? Atmosphere 12:442. 10.3390/atmos12040442

[CR9] Kandemir Y, Doğan NÖ, Yaka E, Pekdemir M, Yılmaz S (2021) Clinical characteristics of neutrophilic, eosinophilic and mixed-type exacerbation phenotypes of COPD. Am J Emerg Med 45:237–241. 10.1016/j.ajem.2020.08.044 33041140 10.1016/j.ajem.2020.08.044

[CR10] Kang HS, Rhee CK, Kim SK, Kim JW, Lee SH, Yoon HK, Ahn JH, Kim YH (2016) Comparison of the clinical characteristics and treatment outcomes of patients requiring hospital admission to treat eosinophilic and neutrophilic exacerbations of COPD. Int J Chron Obstruct Pulmon Dis 11:2467–2473. 10.2147/COPD.S116072 27757029 10.2147/COPD.S116072 PMC5055104

[CR11] Li J, Sun S, Tang R, Qiu H, Huang Q, Mason TG, Tian L (2016) Major air pollutants and risk of COPD exacerbations: a systematic review and meta-analysis. Int J Chron Obstruct Pulmon Dis 11:3079–3091. 10.2147/COPD.S122282 28003742 10.2147/COPD.S122282 PMC5161337

[CR12] Liu K, Hua S, Song L (2022) PM2.5 exposure and asthma development: the key role of oxidative stress. Oxid Med Cell Longev 2022:3618806. 10.1155/2022/3618806 35419163 10.1155/2022/3618806 PMC9001082

[CR13] Narendra DK, Hanania NA (2019) Targeting IL-5 in COPD. Int J Chron Obstruct Pulmon Dis 14:1045–1051. 10.2147/COPD.S155306 31190789 10.2147/COPD.S155306 PMC6529620

[CR14] Nurhussien L, Kang CM, Koutrakis P, Coull BA, Rice MB (2022) Air pollution exposure and daily lung function in chronic obstructive pulmonary disease: effect modification by eosinophil level. Ann Am Thorac Soc 19:728–736. 10.1513/AnnalsATS.202107-846OC 34678126 10.1513/AnnalsATS.202107-846OC PMC9116346

[CR15] Saeed MS, Denoncourt CM, Chao IA, Schortmann S, Nassikas NJ, Synn AJ, Koutrakis P, Coull BA, Kang CM, Wolfson JM, Ferguson ST, Rebuli ME, Jaspers I, Liu JP, Greco KF, Phipatanakul W, Rice MB (2024) Protocol for the air purification for eosinophilic COPD study (APECS): a randomised controlled trial of home air filtration by HEPA. BMJ Open 14:e074655. 10.1136/bmjopen-2023-074655 10.1136/bmjopen-2023-074655 PMC10806745 38238060

[CR16] Şancı E, Özbek AE, Alkan F, Kılınç H, Halhallı HC (2023) Comparison of hematological phenotypes of COPD exacerbations in hospitalized patients after emergency department admission. Istanbul Med J 24:112–115. 10.4274/imj.galenos.2023.09334

[CR17] Tseng CM, Chen YT, Ou SM, Hsiao YH, Li SY, Wang SJ, Yang AC, Chen TJ, Perng DW (2013) The effect of cold temperature on increased exacerbation of chronic obstructive pulmonary disease: a nationwide study. PLoS ONE 8:e57066. 10.1371/journal.pone.0057066 23554858 10.1371/journal.pone.0057066 PMC3598847

[CR18] World Health Organization (2021) WHO global air quality guidelines: particulate matter (PM₂.₅ and PM₁₀), ozone, nitrogen dioxide, sulfur dioxide and carbon monoxide. World Health Organization, Geneva 34662007

