# Supplementary figures and images for: Effects of air pollution and meteorological factors on hematological exacerbation phenotypes in patients presenting to the emergency department with COPD exacerbation
Source: Int J Biometeorol. 2026 Jun 9;70(6):185. doi: 10.1007/s00484-026-03247-9 (PMC13249681; doi:10.1007/s00484-026-03247-9)

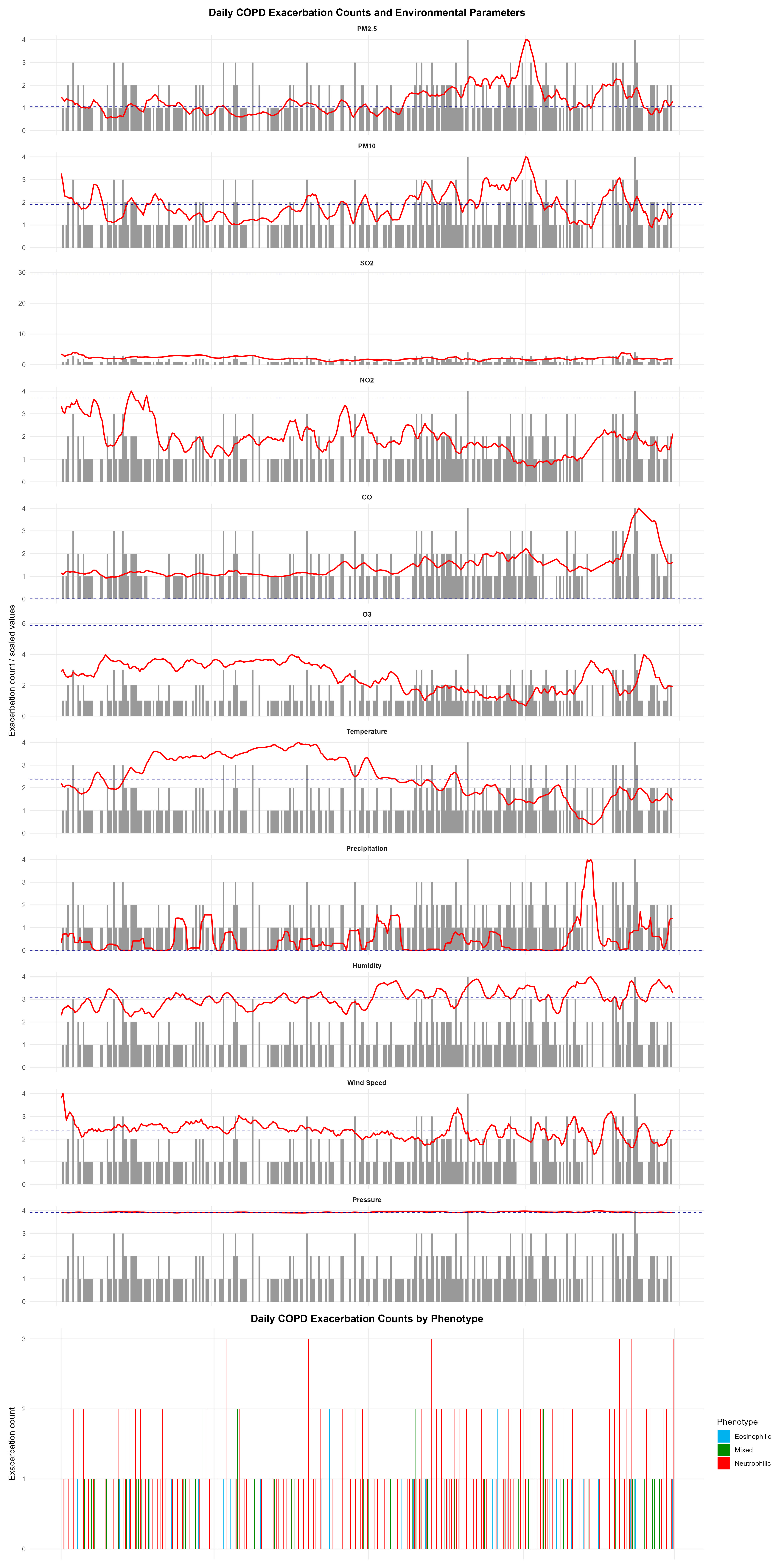

Supplement: Supplementary file 1 — Supplementary Material 1: Daily COPD exacerbation counts (gray bars) plotted alongside the time series of all environmental parameters (red lines, smoothed values; blue dashed lines, WHO or reference thresholds where applicable) over the study period: PM₂.₅, PM₁₀, SO₂, NO₂, CO, O₃, temperature, precipitation, humidity, wind speed, and atmospheric pressure. The bottom panel shows the temporal distribution of daily exacerbation counts colored by hematological phenotype [file 484_2026_3247_MOESM1_ESM.jpeg]

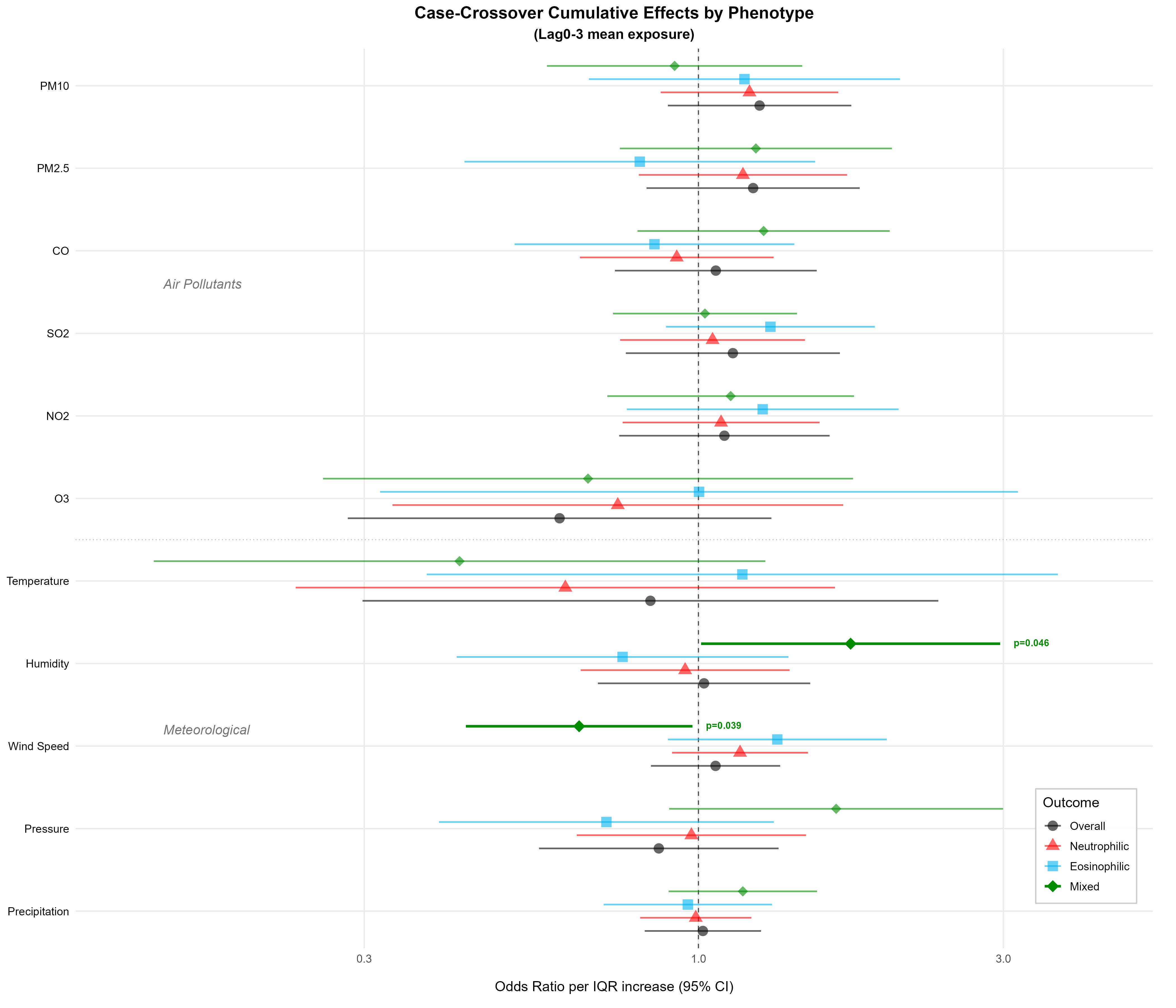

Supplement: Supplementary file 2 — Supplementary Material 2:Cumulative effects (lag 0–3) of environmental parameters on COPD exacerbation counts by hematological phenotype, derived from time-stratified case-crossover analyses. Odds ratios (OR) per IQR increase are shown with 95% confidence intervals for the overall cohort and three phenotypes. Statistically significant associations (p < 0.05) are highlighted with annotated p values [file 484_2026_3247_MOESM2_ESM.jpeg]

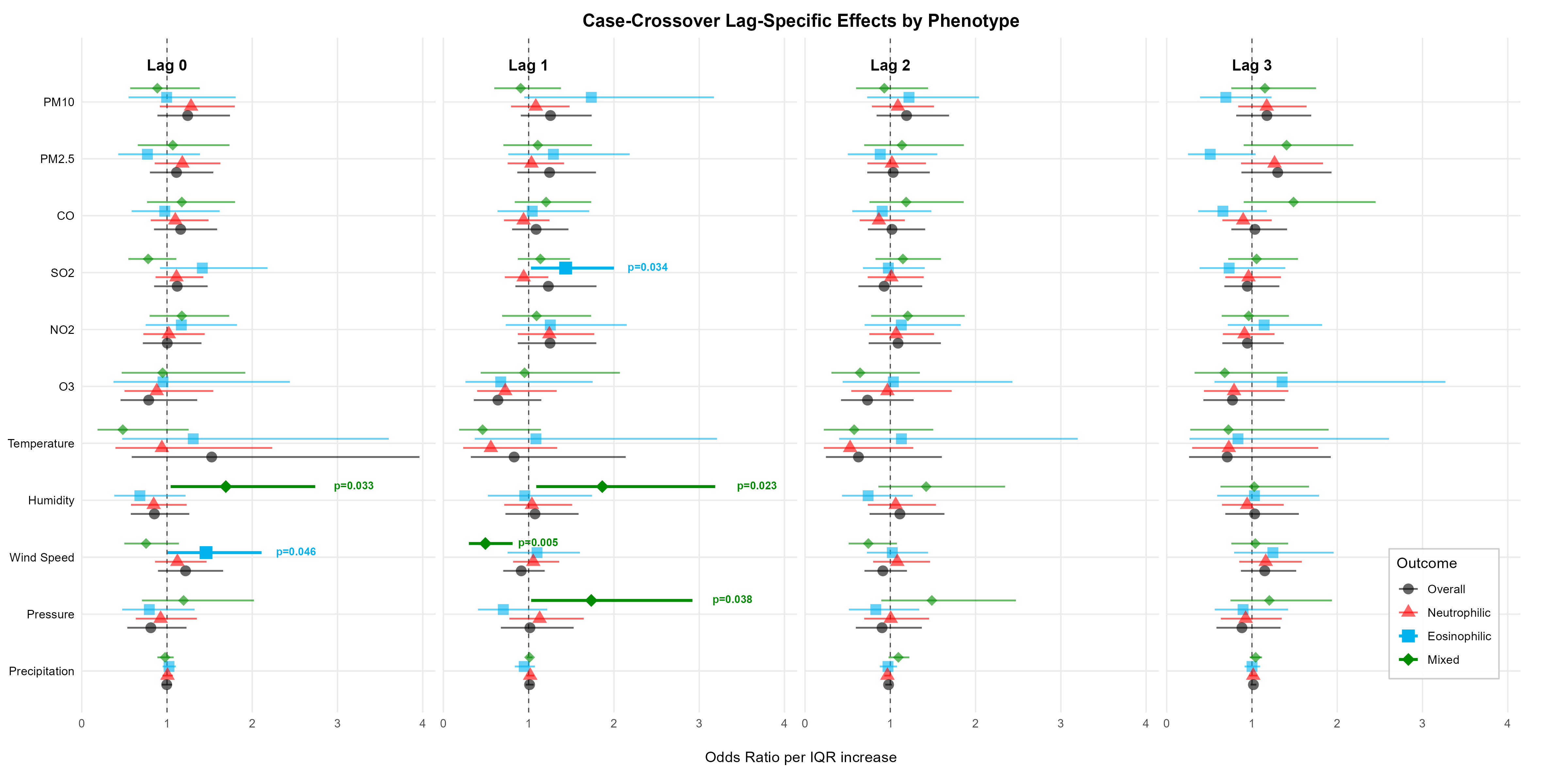

Supplement: Supplementary file 3 — Supplementary Material 3: Lag-specific effects of environmental parameters on COPD exacerbation counts by hematological phenotype, derived from time-stratified case-crossover analyses. Each panel shows odds ratios (OR) per IQR increase with 95% confidence intervals at lag 0, 1, 2, and 3. Statistically significant associations (p < 0.05) are highlighted with annotated p values [file 484_2026_3247_MOESM3_ESM.jpeg]
