# Supplementary material for: Effects of air pollution and meteorological factors on hematological exacerbation phenotypes in patients presenting to the emergency department with COPD exacerbation
Source: Int J Biometeorol. 2026 Jun 9;70(6):185. doi: 10.1007/s00484-026-03247-9 (PMC13249681; doi:10.1007/s00484-026-03247-9)
Supplement: Supplementary file 4 — Supplementary Material 4 [file 484_2026_3247_MOESM4_ESM.docx]

**SUPPLEMENTARY TABLES**

**Supplementary Table 1. Descriptive statistics of environmental parameters during the study period (n = 365 days)**

| **Parameter** | **Unit** | **Median** | **IQR (25th–75th)** | **Min** | **Max** | **WHO AQG limit** | **Days exceeding limit** |
| --- | --- | --- | --- | --- | --- | --- | --- |
| **PM₁₀** | μg/m³ | 40.4 | 27.4–57.0 | 11.1 | 91.0 | 45 μg/m³ | 155 (42.5%) |
| **PM₂.₅** | μg/m³ | 18.5 | 12.0–27.7 | 4.5 | 54.6 | 15 μg/m³ | 202 (55.3%) |
| **SO₂** | μg/m³ | 2.8 | 2.0–3.8 | 0.5 | 11.2 | 40 μg/m³ | 0 (0.0%) |
| **NO₂** | μg/m³ | 22.4 | 16.4–31.2 | 5.1 | 68.7 | 25 μg/m³ | 22 (6.0%) |
| **CO** | μg/m³ | 512 | 385–680 | 180 | 1420 | 4000 μg/m³ | 0 (0.0%) |
| **O₃** | μg/m³ | 41.8 | 28.9–57.0 | 5.3 | 93.0 | 100 μg/m³ | 0 (0.0%) |
| **Temperature** | °C | 17.3 | 10.1–24.2 | 0.2 | 32.5 | — | — |
| **Relative humidity** | % | 72.1 | 60.8–82.4 | 31.0 | 97.0 | — | — |
| **Wind speed** | m/s | 1.4 | 1.2–1.7 | 0.5 | 3.8 | — | — |
| **Atmospheric pressure** | hPa | 1008 | 1003–1012 | 982 | 1026 | — | — |
| **Precipitation** | kg/m² | 0.0 | 0.0–1.1 | 0.0 | 38.4 | — | — |

WHO: World Health Organization; AQG: Air Quality Guidelines (2021); IQR: interquartile range; PM: particulate matter; SO₂: sulfur dioxide; NO₂: nitrogen dioxide; CO: carbon monoxide; O₃: ozone.

Days exceeding limit: number of days exceeding the WHO AQG short-term level. "—" indicates parameters without a WHO short-term limit.

**Supplementary Table 2A. Sensitivity analysis — time-stratified case-crossover design: lag-specific odds ratios for air pollutants (per IQR increase, 95% CI)**

| **Pollutant** | **Lag** | **Overall COPD OR (95% CI)** | **Eosinophilic OR (95% CI)** | **Neutrophilic OR (95% CI)** | **Mixed-type OR (95% CI)** |
| --- | --- | --- | --- | --- | --- |
| **PM₁₀** | Lag 0 | 1.24 (0.89–1.74) | 1.00 (0.55–1.81) | 1.28 (0.92–1.79) | 0.89 (0.57–1.38) |
|  | Lag 1 | 1.26 (0.91–1.74) | 1.73 (0.95–3.17) | 1.08 (0.79–1.48) | 0.91 (0.60–1.38) |
|  | Lag 2 | 1.19 (0.84–1.69) | 1.22 (0.73–2.04) | 1.09 (0.78–1.51) | 0.93 (0.60–1.44) |
|  | Lag 3 | 1.18 (0.82–1.70) | 0.69 (0.39–1.23) | 1.17 (0.84–1.64) | 1.15 (0.76–1.75) |
|  | ***Cumulative*** | ***1.25 (0.90–1.73)*** | ***1.18 (0.67–2.07)*** | ***1.20 (0.87–1.66)*** | ***0.92 (0.58–1.45)*** |
| **PM₂.₅** | Lag 0 | 1.11 (0.80–1.54) | 0.77 (0.43–1.39) | 1.18 (0.86–1.63) | 1.07 (0.66–1.73) |
|  | Lag 1 | 1.25 (0.87–1.79) | 1.29 (0.76–2.19) | 1.03 (0.75–1.42) | 1.11 (0.70–1.74) |
|  | Lag 2 | 1.03 (0.73–1.46) | 0.88 (0.50–1.55) | 1.02 (0.73–1.42) | 1.14 (0.69–1.86) |
|  | Lag 3 | 1.30 (0.88–1.93) | 0.51 (0.25–1.04) | 1.26 (0.87–1.83) | 1.41 (0.90–2.19) |
|  | ***Cumulative*** | ***1.22 (0.83–1.79)*** | ***0.81 (0.43–1.52)*** | ***1.17 (0.81–1.71)*** | ***1.23 (0.75–2.01)*** |
| **CO** | Lag 0 | 1.16 (0.85–1.59) | 0.97 (0.59–1.62) | 1.10 (0.81–1.49) | 1.17 (0.77–1.80) |
|  | Lag 1 | 1.09 (0.81–1.47) | 1.04 (0.63–1.71) | 0.94 (0.71–1.24) | 1.20 (0.84–1.73) |
|  | Lag 2 | 1.02 (0.74–1.41) | 0.91 (0.55–1.48) | 0.87 (0.64–1.17) | 1.19 (0.76–1.86) |
|  | Lag 3 | 1.03 (0.76–1.41) | 0.66 (0.37–1.17) | 0.90 (0.65–1.23) | 1.49 (0.90–2.45) |
|  | ***Cumulative*** | ***1.06 (0.74–1.53)*** | ***0.85 (0.52–1.41)*** | ***0.92 (0.65–1.31)*** | ***1.26 (0.80–1.99)*** |
| **SO₂** | Lag 0 | 1.12 (0.85–1.48) | 1.41 (0.92–2.18) | 1.11 (0.87–1.43) | 0.78 (0.55–1.11) |
|  | Lag 1 | 1.23 (0.84–1.80) | **1.43 (1.03–2.00)** | 0.94 (0.72–1.23) | 1.14 (0.87–1.48) |
|  | Lag 2 | 0.93 (0.63–1.37) | 0.98 (0.68–1.40) | 1.01 (0.73–1.39) | 1.15 (0.83–1.59) |
|  | Lag 3 | 0.95 (0.68–1.32) | 0.73 (0.39–1.39) | 0.96 (0.69–1.34) | 1.05 (0.72–1.54) |
|  | ***Cumulative*** | ***1.13 (0.77–1.66)*** | ***1.30 (0.89–1.89)*** | ***1.05 (0.75–1.47)*** | ***1.02 (0.73–1.43)*** |
| **NO₂** | Lag 0 | 1.00 (0.72–1.40) | 1.17 (0.75–1.82) | 1.02 (0.72–1.44) | 1.17 (0.80–1.73) |
|  | Lag 1 | 1.25 (0.87–1.79) | 1.25 (0.73–2.15) | 1.24 (0.87–1.77) | 1.09 (0.69–1.73) |
|  | Lag 2 | 1.09 (0.75–1.59) | 1.13 (0.70–1.83) | 1.07 (0.75–1.51) | 1.21 (0.78–1.87) |
|  | Lag 3 | 0.95 (0.65–1.37) | 1.14 (0.72–1.82) | 0.91 (0.66–1.26) | 0.96 (0.65–1.43) |
|  | ***Cumulative*** | ***1.10 (0.75–1.60)*** | ***1.26 (0.77–2.06)*** | ***1.08 (0.76–1.55)*** | ***1.12 (0.72–1.75)*** |
| **O₃** | Lag 0 | 0.79 (0.46–1.35) | 0.95 (0.37–2.44) | 0.88 (0.50–1.54) | 0.95 (0.47–1.92) |
|  | Lag 1 | 0.64 (0.36–1.15) | 0.67 (0.26–1.75) | 0.73 (0.40–1.33) | 0.95 (0.44–2.07) |
|  | Lag 2 | 0.73 (0.42–1.27) | 1.04 (0.44–2.43) | 0.97 (0.54–1.72) | 0.65 (0.31–1.35) |
|  | Lag 3 | 0.77 (0.43–1.38) | 1.35 (0.56–3.27) | 0.79 (0.44–1.43) | 0.68 (0.33–1.42) |
|  | ***Cumulative*** | ***0.61 (0.28–1.30)*** | ***1.00 (0.32–3.16)*** | ***0.75 (0.33–1.68)*** | ***0.67 (0.26–1.75)*** |

OR: odds ratio; CI: confidence interval; IQR: interquartile range (calculated per phenotype reference group). Conditional logistic regression was used; each case was matched to control days within the same calendar month and on the same day of the week.

Bold values are statistically significant at p < 0.05. "Cumulative" corresponds to the average exposure across lag 0–3 days.

**Supplementary Table 2B. Sensitivity analysis — time-stratified case-crossover design: lag-specific odds ratios for meteorological parameters (per IQR increase, 95% CI)**

| **Parameter** | **Lag** | **Overall COPD OR (95% CI)** | **Eosinophilic OR (95% CI)** | **Neutrophilic OR (95% CI)** | **Mixed-type OR (95% CI)** |
| --- | --- | --- | --- | --- | --- |
| **Temperature** | Lag 0 | 1.52 (0.59–3.96) | 1.31 (0.47–3.60) | 0.94 (0.40–2.23) | 0.48 (0.19–1.25) |
|  | Lag 1 | 0.83 (0.32–2.14) | 1.09 (0.37–3.21) | 0.56 (0.23–1.33) | 0.46 (0.18–1.14) |
|  | Lag 2 | 0.63 (0.24–1.60) | 1.13 (0.40–3.20) | 0.53 (0.22–1.27) | 0.58 (0.22–1.50) |
|  | Lag 3 | 0.71 (0.26–1.92) | 0.84 (0.27–2.61) | 0.73 (0.30–1.78) | 0.72 (0.28–1.90) |
|  | ***Cumulative*** | ***0.84 (0.30–2.37)*** | ***1.17 (0.38–3.65)*** | ***0.62 (0.23–1.64)*** | ***0.42 (0.14–1.27)*** |
| **Humidity** | Lag 0 | 0.85 (0.58–1.26) | 0.68 (0.38–1.22) | 0.84 (0.58–1.23) | **1.69 (1.04–2.74)** |
|  | Lag 1 | 1.07 (0.73–1.59) | 0.95 (0.52–1.74) | 1.04 (0.71–1.51) | **1.86 (1.09–3.19)** |
|  | Lag 2 | 1.11 (0.76–1.64) | 0.74 (0.43–1.26) | 1.06 (0.74–1.53) | 1.42 (0.86–2.35) |
|  | Lag 3 | 1.03 (0.69–1.55) | 1.03 (0.59–1.79) | 0.94 (0.65–1.37) | 1.03 (0.63–1.67) |
|  | ***Cumulative*** | ***1.02 (0.70–1.50)*** | ***0.76 (0.42–1.38)*** | ***0.95 (0.65–1.39)*** | ***1.73 (1.01–2.97)*** |
| **Wind speed** | Lag 0 | 1.22 (0.89–1.66) | **1.46 (1.01–2.11)** | 1.12 (0.86–1.46) | 0.75 (0.50–1.14) |
|  | Lag 1 | 0.91 (0.70–1.19) | 1.10 (0.75–1.60) | 1.05 (0.82–1.36) | **0.49 (0.30–0.81)** |
|  | Lag 2 | 0.91 (0.70–1.20) | 1.02 (0.73–1.44) | 1.08 (0.80–1.47) | 0.74 (0.51–1.08) |
|  | Lag 3 | 1.15 (0.87–1.52) | 1.24 (0.79–1.96) | 1.16 (0.85–1.58) | 1.04 (0.76–1.42) |
|  | ***Cumulative*** | ***1.06 (0.84–1.34)*** | ***1.33 (0.90–1.97)*** | ***1.16 (0.91–1.48)*** | ***0.65 (0.43–0.98)*** |
| **Atmospheric pressure** | Lag 0 | 0.81 (0.53–1.23) | 0.79 (0.47–1.32) | 0.92 (0.63–1.35) | 1.19 (0.71–2.02) |
|  | Lag 1 | 1.01 (0.67–1.53) | 0.70 (0.41–1.22) | 1.13 (0.77–1.65) | **1.73 (1.03–2.92)** |
|  | Lag 2 | 0.90 (0.60–1.37) | 0.83 (0.51–1.34) | 1.00 (0.69–1.46) | 1.49 (0.89–2.47) |
|  | Lag 3 | 0.88 (0.58–1.33) | 0.90 (0.56–1.42) | 0.93 (0.63–1.35) | 1.20 (0.75–1.94) |
|  | ***Cumulative*** | ***0.87 (0.56–1.33)*** | ***0.72 (0.39–1.31)*** | ***0.97 (0.65–1.47)*** | ***1.64 (0.90–3.00)*** |
| **Precipitation** | Lag 0 | 1.00 (0.94–1.06) | 1.02 (0.95–1.10) | 1.01 (0.95–1.07) | 0.98 (0.89–1.08) |
|  | Lag 1 | 1.01 (0.96–1.06) | 0.95 (0.84–1.07) | 1.02 (0.97–1.06) | 1.01 (0.98–1.05) |
|  | Lag 2 | 0.98 (0.94–1.02) | 0.97 (0.88–1.08) | 0.97 (0.93–1.00) | 1.09 (0.98–1.22) |
|  | Lag 3 | 1.02 (0.98–1.05) | 1.00 (0.91–1.10) | 1.01 (0.97–1.06) | 1.04 (0.97–1.12) |
|  | ***Cumulative*** | ***1.02 (0.82–1.25)*** | ***0.96 (0.71–1.30)*** | ***0.99 (0.81–1.21)*** | ***1.17 (0.90–1.53)*** |

OR: odds ratio; CI: confidence interval; IQR: interquartile range (calculated per phenotype reference group). Conditional logistic regression was used; each case was matched to control days within the same calendar month and on the same day of the week.

Bold values are statistically significant at p < 0.05. "Cumulative" corresponds to the average exposure across lag 0–3 days.

**Supplementary Table 3. DLNM model specifications and collinearity diagnostics**

**3A. Technical specifications of the model**

| **Model component** | **Specification** |
| --- | --- |
| **Response variable** | Daily exacerbation count; quasi-Poisson family with log link function |
| **Exposure–response function** | Linear (linear cross-basis) |
| **Lag function** | Natural cubic spline, 2 df, maximum lag of 3 days |
| **Time-trend control** | Natural cubic spline, 7 df/year (long-term trend and seasonality) |
| **Day of week** | Categorical covariate (six dummy variables) |
| **Pollutant modeling** | Single-pollutant approach; each parameter analyzed in a separate model |
| **Effect estimation** | RR per IQR increase (95% CI); lag-specific and cumulative |
| **Software** | R 4.3.0 (dlnm, mgcv, survival, dplyr, ggplot2 packages); IBM SPSS Statistics 29.0 |

**3B. Collinearity diagnostics (Lag-0 parameters in the single-pollutant model context)**

| **Parameter** | **VIF** | **Pearson r (with PM₁₀)** | **Interpretation** |
| --- | --- | --- | --- |
| **PM₁₀** | 3.98 | 1.00 (reference) | Acceptable |
| **PM₂.₅** | 4.27 | 0.82 | High correlation — modeled separately |
| **CO** | 1.35 | 0.54 | Acceptable |
| **O₃** | 1.86 | −0.58 | Inverse correlation — modeled separately |
| **SO₂** | 1.16 | 0.31 | Acceptable |
| **NO₂** | 1.42 | 0.47 | Acceptable |
| **Temperature** | 1.60 | −0.43 | Acceptable |
| **Relative humidity** | 1.38 | −0.21 | Acceptable |

VIF: variance inflation factor. Multicollinearity was considered acceptable when VIF < 5. Because all pollutants were analyzed in separate single-pollutant DLNM models, high inter-pollutant correlations do not affect the model estimates.

Pearson r values were calculated at lag 0; negative values indicate an inverse correlation.

DLNM: distributed lag non-linear model; RR: relative risk; CI: confidence interval; IQR: interquartile range; df: degrees of freedom.
